# Prognostic Value of MRS Metabolites in Postoperative Irradiated High Grade Gliomas

**DOI:** 10.1155/2015/341042

**Published:** 2015-08-03

**Authors:** Maria Tolia, Dimitrios Verganelakis, Nikolaos Tsoukalas, George Kyrgias, Matilda Papathanasiou, Eftichia Mosa, Ioannis Kokakis, John R. Kouvaris, George Pissakas, Kyriaki Pistevou-Gombaki, Nikolaos Kelekis, Vasileios Kouloulias

**Affiliations:** ^1^2nd Department of Radiology, Radiation Oncology Unit, Medical School, Attikon University Hospital, 1 Rimini Street, Haidari, 12462 Athens, Greece; ^2^Encefalos Radiology Center, 3 Rizareiou & Chrisostomou Smirnis Street, Chalandri, 15233 Athens, Greece; ^3^Medical Oncology Unit, 401 General Army Hospital, 10-12 Gennimata Street, Ampelokipi, 11524 Athens, Greece; ^4^School of Health Sciences, Faculty of Medicine, Department of Radiotherapy, University of Thessaly, Biopolis, 41110 Larissa, Greece; ^5^1st Department of Radiology, Radiation Oncology Unit, Medical School, Aretaieion University Hospital, Vas Sofias 104 Avenue, 11521 Athens, Greece; ^6^Radiotherapy Department, Alexandra Hospital, 11521 Athens, Greece; ^7^Department of Radiation Oncology, AHEPA Hospital, Aristotle University of Thessaloniki, 1 Kyriakidi Street, 54636 Thessaloniki, Greece

## Abstract

*Purpose*. We studied the prognostic significance of Magnetic Resonance Spectroscopy (MRS) in operated high grade gliomas. *Materials and Methods*. Twelve patients were treated with radiotherapy and Temozolomide. The MRS data were taken four weeks after operation (before radiotherapy) and every six months after the completion of RT. The N-acetyl aspartate, choline, creatine, and myo-inositol parameters were quantified, analyzed, and correlated to recurrence-free survival (RFS). *Results*. The median RFS was 26.06 months. RFS was significantly worse in elderly patients (*P* = 0.001) along with the higher choline/creatine ratios at either baseline (*P* = 0.003) or six months post Radiotherapy (*P* = 0.042). Median RFS was 23 months in high choline/creatine levels ≥2 at 6 months after radiotherapy and 11 months for those with <2 choline/creatine levels. There was a significant correlation of maximum difference of choline/creatine ratio with RFS (rho = 0.64, *P* = 0.045). *Conclusion*. Age and choline/creatine ratio are strong independent prognostic factors in high grade gliomas.

## 1. Introduction

High grade gliomas, anaplastic astrocytoma (grade III) and glioblastoma (grade IV) classified according to the World Health Organization (WHO), represent the commonest primary adult cerebral neoplasms. They account for approximately 7% and 54% of all gliomas [1]. Only 30% of patients survive more than 1 year and less than 5% beyond 5 years. The 5-year survival rate for anaplastic astrocytoma is 27% [2].

Magnetic Resonance Spectroscopy (MRS) can offer biochemical data from the different volumes of interest in tissues. It can provide metabolic information on tumor activity regarding rate of growth, heterogeneity, and extension and permits a noninvasive categorization of brain tumors. It is useful in grading tumors and can help in treatment strategies (as surgery, radiotherapy, chemotherapy, angiogenesis inhibitors, etc.), in radiation treatment planning delineation, in evaluating response to treatment, and in discriminating tumor from radiation necrosis. MRS imaging enables the assessment of tissue metabolites such as choline (Cho), creatine (Cr), N-acetylaspartate (NAA), and myo-inositol (MI). Cho is a marker of phospholipid turnover and cellular density and it is increased in all gliomas [3]. N-Acetyl aspartate (NAA) is found in neuronal cell types and oligodendrocytes. Cho represents the precursor of phosphatidyl Cho (phospholipid of cell membrane). In malignant tumors there are higher levels of Cho because of the increased cell membrane turnover. The values of Cho are higher in grades III and IV than in grade II astrocytomas and it is relatively diminished when necrosis is present [4]. In brain tumors there are low levels of NAA and higher Cho levels because of the neuronal destruction, the high proliferation rate, and increased cell membrane turnover. Cr is a high-energy compound located in the mitochondria and serves as a marker for cellular energy metabolism. The ratios of Cho/NAA and Cho/Cr are higher in high grade gliomas, compared with low grade gliomas [5].

Many prognostic factors have been studied as influencing the outcome of these aggressive tumors [6–12]. Advanced age is inversely related to survival (*P* < 0.0001) [6]. Lobar tumor location, radiation therapy (RT) dose 5,000–6,000 cGy, Karnofsky performance status (KPS) at presentation ≥70, and a normal level of consciousness before biopsy are considered good prognostic factors [7]. Age (*P* = 0.027), log10 of epidermal growth factor receptor (EGFR) (*P* = 0.025), and labeling index (LI) measured by tritiated thymidine incorporation (*P* = 0.0019) were significant continuous variables and the survival was found to be shorter when the covariable increased [8]. Frontally located tumors were found to have longer median survival time and higher 1- and 2-year survival rates compared to tumors in other locations (101 versus 47 weeks, resp.; 76% and 44% versus 37% and 2.5%, resp.; *P* = 0.00001). Progression-free survival at 1 year was higher in the radically resected group than in the group that was biopsied (20% versus 0%, resp.; *P* < 0.001) [9]. Microvessel density of grade of 3+ or 4+ was found to correlate with shorter survival time than microvessel density grade of 1+ or 2+ (*P* = 0.0022) [10]. A statistically significant improvement in survival was associated with increasing total radiation dose to the tumor bed (*P* < 0.001) without additional benefit demonstrated for doses greater than 60 Gy [11]. Among patients with KPS ≥ 70 and age < 50 years, median survival was 57 weeks if the corpus callosum was involved (35% 2-year survival) and 105 weeks if the corpus callosum was not involved (56% 2-year survival) [12].

The aim of this study was to determine whether MRS can be used for prognosis of recurrence in postoperative irradiated high grade gliomas and to correlate MRS metabolites with RFS.

## 2. Materials and Methods

Twelve patients (six females and six males) with a diagnosis of high grade glioma participated in the present study. All participating patients firmed the informed consent and etic committee approval was not needed. The patients' characteristics are shown in Table 1. The median age was 51 years (range: 29–72 years). All patients presented with central nervous system symptoms and were assessed with brain MRI that demonstrated a lesion compatible with brain tumor. All patients underwent surgery and biopsy confirmed a high grade glioma grades III-IV, according to the World Health Organization (WHO) classification. Six patients were diagnosed with a glioma grade III and 6 with a glioblastoma multiforme. Patients were evaluated with MRS before the delivery of external beam three-dimensional conformal radiotherapy (3D-CRT). We excluded gliomas located in the brainstem and patients with Karnofsky performance status <80.

All patients underwent an MRS at baseline before the initialization of RT and six months after irradiation. All the MRI examinations were performed on a 1.5 Tesla system (General Electric, Signa HDxt, Winsconsin, USA). The MRI protocol included the following pulse sequences: axial T2 flair (TE: 112 ms, TR: 9002 ms, TI: 2250 ms, 5 mm slice thickness and 1.5 mm gap, and 320 × 224 matrix), coronal diffusion (TE: 100 ms, TR: 4500 ms, 5 mm slice thickness and 1.5 mm gap, and 128 × 128 matrix), and axial T2 multiecho (TE: varying, TR: 675 ms, 5 mm of slice thickness and 1.5 mm gap, and 256 × 160 matrix). These pulse sequences help towards the differentiation of the tumor as well as the placement of the single-voxel and the 3D slab (i.e., active tumor volume and not edema). The MRS pulse sequences were single-voxel PRESS at TE: 35 ms and 135 ms (of variable voxel sizes which were normalized for comparison reasons, TR: 1500 ms, NEX: 8) and 3-dimensional PRESS at TE: 135 (TR: 1000 ms, of variable thickness and spacing between patients with typical values of the order of 48.5 thickness and 8.1 mm spacing, 10 × 10 matrix, and NEX: 0.80).

Each patient underwent a virtual CT-simulation, in the supine position, using dedicated devices. Patients were fixed in a custom-designed immobilization device and were simulated and treated in the supine position. The patients were scanned with 5 mm slice thickness in simulation CT scan and the CT datasets were transferred to the Prosoma Virtual Simulation and Contouring System through the DICOM network. The following structures were delineated as organs at risk (OARs): optic chiasm, optic nerves, brainstem, eyes, and lenses. The Clinical Target Volume (CTV) was delineated using preoperative and postoperative MRI and postoperative MRS. The surgical cavity, the areas of contrast enhancement, and T2 flair signal abnormality expanded by 2-3 cm for subdiagnostic microscopic infiltration constituted the CTV. A margin of 5 mm to the CTV was added to generate the Planning Target Volume (PTV). Contours were edited to exclude air, bone, and brain parenchyma if possible.

RT was administered within 4–6 weeks of the surgery. The patients were all treated with adjuvant fractionated external beam 3D-CRT. A total dose of 60 Gy was delivered in 30 daily fractions (2 Gy/fraction) [13]. Fields were reduced for the last phase of the treatment as boost. The prescription dose was defined for the 95% isodose of the PTV. In particular, 95% of the PTV should have been covered within 95%–110% of the prescribed dose. The treatment planning was performed in the Eclipse (Varian Medical Systems, United States) treatment planning system (TPS). The photon beam energies used were 6 MV, using a 2100C Varian linear accelerator. The beam arrangement consisted of beams, where the beam angles, apertures, weights, and dynamic wedges were optimized by standard, forward planning. Partial wedging or dynamic (multileaf collimator (MLC)) was employed to improve dose homogeneity.

The target volumes and organs at risk were elaborated with the beam's eye view (BEV) technique. For the treatment technique, histograms of the OARs were generated; a number of parameters, including mean, median, and maximum dose, were evaluated.

Concurrent (with RT) 75 mg/m^2^/daily of temozolomide (TMZ) was administered. After RT TMZ dose was 150–200 mg/m^2^ per day for 5 days of a 28-day cycle [13]. The dose was adjusted according to standard hematological toxicity criteria. The intention was to give patients at least 12 cycles (up to 24) of TMZ, and treatment was continued until we observed disease progression or unacceptable toxicity.

During radiation treatment the patients were monitored every week. Posttreatment management included adjuvant endocrine therapy according to the National Comprehensive Cancer Network Guidelines. After completion of treatment, the patients were evaluated by a radiation oncologist every 3 mo.

The duration of RFS for patients with high grade gliomas was measured from the time of diagnosis to the time of recurrence (viable tumor on magnetic resonance imaging). To evaluate prognostic values, we performed univariate and multivariate Cox-regression survival analysis in terms of MRS parameters such as Cho, Cr, NAA, MI, NAA/Cr, Cho/Cr, Cho/NAA, and MI/Cr. Briefly, all factors with *P* < 0.05 on univariate analysis were entered into the model and the model was refit in a stepwise fashion after the sequential removal of nonsignificant factors. The process was stopped when only significant (*P* < 0.05) factors remained. Entering the significant factors sequentially and checking for and possibly removing factors that became nonsignificant confirmed the model. The correlation of RFS with the maximum difference of Cho/Cr ratio was performed with the Spearman rho test. Kaplan Meier curve and log-rank test were performed for RFS related to either <2 or ≥2 of Cho/Cr ratio in two cases: for baseline and 6 months after RT Cho/Cr ratio. The median value of Cho/Cr ratio was chosen as the cut-point for the comparative analysis of RFS related to Cho/Cr ratio. Statistical significance was accepted at the *P* < 0.05 level. All the analysis was performed with the SPSS software v.10 (IL, USA).

## 3. Results

**Figure 1:**
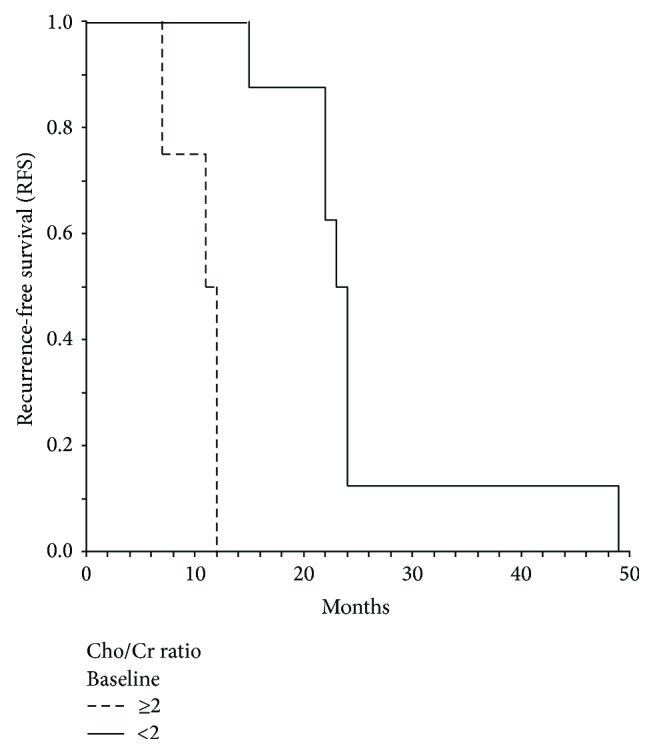
Kaplan-Meier curve for recurrence-free survival (RFS) in terms of Cho/Cr ratio ≥2 versus <2 (baseline). Median RFS 23 (SE = 1) versus 11 (SE = 2), log-rank *P* = 0.0004.

**Figure 2:**
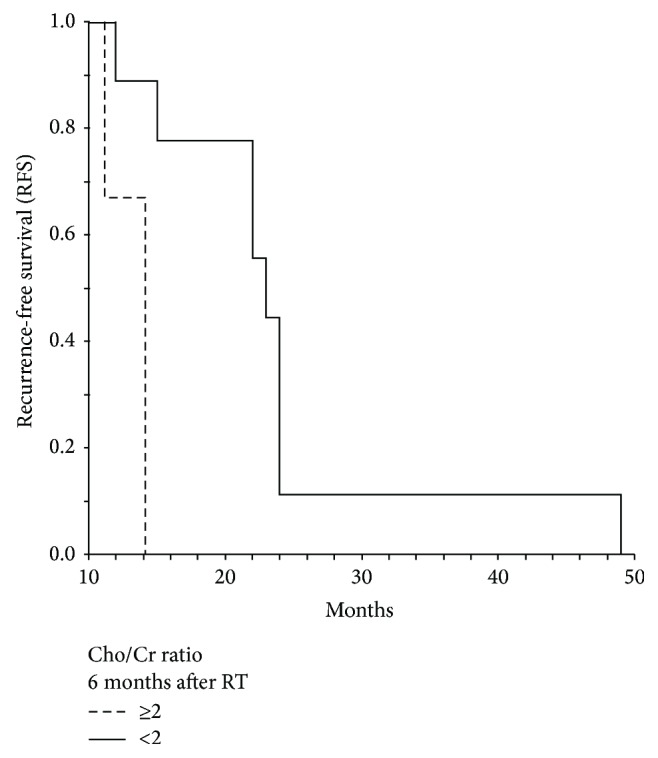
Kaplan-Meier curve for recurrence-free survival (RFS) in terms of Cho/Cr ratio ≥2 versus <2 (6 months after RT). Median RFS 23 (SE = 2) versus 11 (SE = 1), log-rank *P* = 0.045.

**Figure 3:**
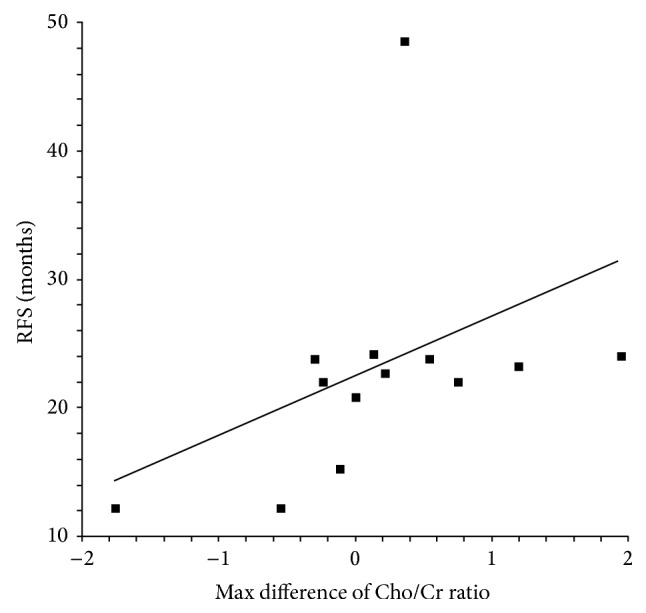
Recurrence-free survival (RFS) correlated with max difference of Cho/Cr ratio with baseline (Spearman rho = 0.64, *P* = 0.045).

All patients completed their irradiation schedule. At the most recent follow-up, five patients were alive and seven were dead. Median RFS was 26.06 months. The median overall survival time was thirty-two months. The patients' characteristics along with the RFS and MRS parameters are summarized in Table 1. In univariate analysis, age, NAA, Cho/Cr (baseline), and Cho/Cr at 6 months after RT were significant prognostic factors for RFS. When the above factors were entered into the multivariate model, the NNA lost its prognostic value, while only age and Cho/Cr ratios at baseline and 6 months thereafter had a significant impact top RFS. The other MRS parameters had no significant impact on RFS. The Cox-regression survival analysis for RFS between age and the MRS findings is shown in Table 2. As shown in Figures 1 and 2 median RFS was 23 months for patients with high Cho/Cr levels ≥2 (at baseline and 6 months after RT) and 11 months for those with <2 Cho/Cr levels (log-rank test: *P* = 0.0004 and *P* = 0.045, resp.). There was a significant correlation of maximum difference of Cho/Cr ratio with RFS (rho = 0.64, *P* = 0.045), as shown in Figure 3.

## 4. Discussion

MRS is employed to obtain metabolic information regarding intracranial gliomas. It represents a useful tool that can allow improvement in neoplasia grading, biopsy/therapy guidance, and earlier evaluation of the response to therapy [14]. Regarding the assessment of brain tumours, conventional MRI deals with structural changes. However, recently developed advanced MRI techniques, such as diffusion weighted imaging (DWI), diffusion tensor imaging (DTI), and perfusion imaging, allow further studies of brain tumours delivering more reliable differential tumour diagnosis as well as tumour grading [15]. Briefly, in DWI studies, the majority of tumours demonstrate higher Apparent Diffusion Coefficients (ADC) values than in healthy brains [16]. Furthermore, DTI demonstrates the effects of tumours on the surrounding white matter tracts, who will be either displaced, or infiltrated, or destroyed. Fractional Anisotropy (F.A.) index has lower values in gliomas than in healthy brains due to abnormal brain architecture [15]. Perfusion MRI allows the study of blood supply in tumours via imaging of tumour vascularity either by IV contrast medium injection (Dynamic Susceptibility Contrast Imaging—DSCI, or Dynamic Contrast Enhancement—DCE) or by endogenous contrast mechanism (Arterial Spin Labelling—ASL) [15].

The multivoxel MRS technique, the so-called chemical shift imaging, has advantages compared to the single voxel spectroscopy: it provides simultaneously spectra information over a wider volume of brain where early tumour diffusion can be detected. Additionally, colour maps with respect to various metabolites can be displayed all over the selected 3D slab.

However, in case that Chemical Shift Imaging (CSI) is used then a number of issues have to be considered, such as, the long acquisition which can lead to patients' anxiety which is accompanied by head motion producing spectra deterioration, field inhomogeneities that produce large line widths deteriorating the spectra resolution, chemical shift misregistration and outer volume signal bleed [17]. In general, the single voxel technique is used for metabolites' quantification, whereas CSI is used for metabolites' spatial distribution [18].

Lee et al. [19] studied the pattern of failure of high grade astrocytomas treated with high dose conformal irradiation (70 or 80 Gy) and have shown that 89% of the patients failed with central or in-field recurrences, 8% had a marginal component to the recurrence, and 3% failed outside the high dose region. Oppitz et al. [20] evaluated the three-dimensional tumor regrowth relative to the treated volume which included the preoperative macroscopic tumor and a 2 cm margin. They found that the majority of tumor recurrences were located within the original 90% isodose. They suggested dose escalation to a more restricted volume. Narayana et al. [21] studied the utility of MRS and functional MRI (fMRI) in RT treatment planning. They evaluated 12 patients with MRS and functional imaging before irradiation. The Cho/Cr ratio maps were fused with the MR images and then transferred to treatment planning CT images for target volume delineation. They found that MRS volumes based on Cho/Cr ≥ 3 were 40% larger than MRI-T1 and functional MRI and assisted in beam orientation. Pirzkall et al. [22] studied the role of MRS in RT target delineation. They found that MRS optimized the target volume delineation and can improve local control. All their patients had MRI and MRS preoperatively. Target volumes were delineated on T1 + gadolinium images. MRS parameters were converted in a quantitative index and were displayed as three-dimensional contours. They found that MRS defined metabolically active gliomas extended beyond the T2 volume by approximately 3 cm. Balmaceda et al. [23] assessed the use of MRS in chemotherapy response of low grade gliomas. They found a significant correlation between increased Lac/Cr and Cho/Cr ratios during treatment and a decreased DFS. They concluded that Cho/Cr and Lac/Cr appeared to be reliable biomarkers of tumor progression. Zeng et al. [24] reported that MRS could discriminate postradiation injury from recurrence. They found higher Cho/NAA and Cho/Cr ratios and lower NAA/Cr ratios in recurrent tumors. The Cho/Cr and Cho/NAA ratios were lower in postradiation injury than in normal cerebral tissue. Weybright et al. [25] suggested that MRS parameters can help in diagnostic dilemmas regarding recurrent or residual tumor, treatment-related changes of posterior fossa, or brainstem tumors. They concluded that mean Cho/Cr ratios obtained in recurrent tumor, treatment-related changes, and normal white matter were 2.93, 1.62, and 0.97, respectively, mean Cho/NAA ratios were 4.34, 1.74, and 0.93, and mean NAA/Cr ratios were 0.74, 0.92, and 1.26, respectively. Alexander et al. [26] suggested that values of Cho during a RT course could indicate response to treatment. All patients were examined with MRS at diagnosis, at week 4 of treatment, and 2 months after RT. Patients who had >40% decrease in Cho levels between week 4 and after RT had a worse outcome (*P* = 0.003) and disease progression (*P* = 0.012). Czernicki et al. [27] performed MRS preoperatively and postoperatively at 6 months in high grade glioma patients. An increase in Cho/NAA and decrease in NAA/Cr ratios were associated with a shorter overall survival. In tumor recurrence Cho/NAA and Lac/Cr ratios increased and the NAA/Cr ratio decreased between the two evaluations. Quon et al. [28] performed MRS in high grade gliomas before RT, at week 4 of irradiation, and 2 months after treatment. They noted that a decrease of >40% in Cho levels from week 4 during RT to 2 months after RT had a statistically significant worse OS (9.1 months versus not reached, *P* < 0.001) and PFS (5.8 versus 19.8 months, *P* = 0.0018).

Our analysis indicates a significant correlation between postoperative Cho/Cr levels and prognosis in terms of recurrence-free survival. The survival of patients with high grade gliomas depends mainly on the intrinsic properties of tumor as potent malignancy and response to treatment. The present study examined a probable association between the postsurgery, pre- and post-RT-treatment MRS metabolite values, and disease-free survival. In our study age seemed to be related with a dismal prognosis, in accordance with the literature [29]. However, the most notable finding was that Cho/Cr ratio was associated with RFS.

## 5. Conclusion

This study has assessed the value of preirradiated in vivo MRS parameters in predicting RFS for patients with high grade gliomas, who undergone postoperative RT. With our findings we are sharing our experience concerning the fact that metabolic measures of residual tumor volume can be strongly related with survival, in terms of Cho/Cr ratio. Cho/Cr ratio is reproducible and can be easily incorporated into routine radiological examination, while it may optimize risk stratification and could be target for evaluating future therapies. The main weakness of the present study is the small number of patients. Future studies should further assess the parameters that have been identified in larger populations of patients and evaluate whether they can also be used to assess prognosis at other time points.

## Figures and Tables

**Table 1:** Patient characteristics and descriptive statistics of MRS parameters.

		Range

Age (median)	51	29–72
Sex (male/female)	6/6	
RFS (median)	26.06	7–49
Cr (mean)	4.309 ± 1.251	1.5–6.5
NAA (mean)	3.919 ± 1.466	1.2–6.8
MI (mean)	6.377 ± 2.914	2.4–14.4
NAA/Cr (mean)	1.0423 ± 0.3798	0.4–2.04
MI/Cr (mean)	0.9026 ± 0.3309	0.45–1.88
Cho/Cr (mean)	1.8216 ± 1.148	0.73–5.0

Abbreviations: RFS: Relapse Free Survival Interval; Cho: choline; Cr: creatine; MI: myo-inositol; NAA: N-acetyl aspartate.

**Table 2:** Cox regression survival analysis or RFS with age and MRS parameters. All significant parameters from univariate analysis were entered into the multivariate analysis to create the final model (Wald Chi-square_3_ = 25.03, *P* < 0.001).

Variables	HR (95% CI)	*P*	HR (95% CI)	*P*
	Univariate		Multivariate	
Cho	—	0.87	—	
Cr	—	0.70	—	
NAA	1.23 (1.08, 16.39)	0.045	—	
MI	—	0.81	—	
Cho/Cr (baseline)	2.94 (1.22, 9.51)	0.001	2.38 (1.17, 8.78)	0.003
Cho/Cr (6 months)	1.54 (1.03, 12.76)	0.021	1.29 (1.08, 11.33)	0.042
NAA/Cr	—	0.31	—	
MI/Cr	—	0.72	—	
Age	6.85 (2.57, 11.36)	0.001	5.44 (2.11, 12.26)	0.001

Abbreviations: DFS: Disease Free Survival Interval; Cho: choline; Cr: creatine; MI: myo-inositol; NAA: N-acetyl aspartate.
